# Increasing magnitude of color differences amplifies category effects

**DOI:** 10.1038/s41598-024-64215-0

**Published:** 2024-06-13

**Authors:** Kashi Li, Takehiro Nagai

**Affiliations:** Department of Information and Communications Engineering, Tokyo Institute of Technology, 4259-G2-1 Nagatsuta-cho, Midori-ku, Yokohama, 226-8502 Japan

**Keywords:** Psychology, Human behaviour

## Abstract

Previous studies have identified differences in sensitivity characteristics between color discrimination and perception of suprathreshold color differences. However, it remains highly unclear how color difference sensitivity changes with increasing magnitudes of color difference along various color hues. This study aimed to quantify the sensitivity transition across various magnitudes of color differences and uncover the underlying mechanisms. Color discrimination sensitivities were measured using an adaptive staircase method for 32 isoluminant pedestal colors in the *u'v'* chromaticity diagram. For suprathreshold color differences, we employed the Maximum Likelihood Difference Scaling (MLDS) method to measure sensitivity to various color difference levels for the same 32 colors. Our findings confirmed the differences in sensitivity characteristics between discrimination and suprathreshold color difference perception. Furthermore, we observed increased sensitivities at many color category boundaries in suprathreshold color difference perception. By investigating the relation between the category effects and the color difference size levels through a model simulation, our findings suggest that the influence of color categories on the perception of color differences may become more pronounced as the magnitude of color differences increases.

## Introduction

Perceiving color differences is an essential ability for human beings, as it forms the basis for detecting and recognizing objects. We perceive color differences of various sizes: subtle color differences, known as just noticeable differences (JNDs), larger color differences referred to as suprathreshold color differences, and even larger differences that often cross color category boundaries. The mechanisms underlying these different sizes of color difference perception have been extensively investigated through psychophysical studies. Specifically, a considerable number of experiments have been conducted on color discrimination, leading to a clearer understanding of the color representations that underlie this discrimination process^1–3^.

One of the fundamental questions in color difference perception is whether the same underlying mechanisms govern perceptual color differences across varying magnitudes. From a classical perspective, Weber–Fechner’s law assumes that the perception of large color differences arises from the integration of JNDs. In other words, this law assumes that the perceptions of color differences of various sizes share the same color representation mechanisms. Hillis and Brainard^4^ directly tested this assumption by conducting color discrimination and color appearance experiments focusing on the effects of the background color. The color discrimination experiment was conducted on several backgrounds with different colors. The color appearance experiment was conducted using an asymmetric color-matching task between a chromatic background and a gray background. They proposed a model based on a variant of Fechner’s proposal that explains both color discrimination and color appearance. In this model, the color appearances of two stimuli matched when the model produced the same responses for them, and color discrimination sensitivity corresponded to the response slope of the model. This model reasonably accounted for the effects of background colors on the performances of both tasks, suggesting that color discrimination and appearance are governed by the same underlying mechanism.

On the other hand, some studies suggested discrimination sensitivities and detection of suprathreshold color differences might involve different underlying mechanisms. For instance, it has been established that contrast perception exhibits distinct behaviors at threshold and suprathreshold levels^5^. The luminance contrast sensitivity functions for spatial frequencies exhibit a band-pass shape with maximum sensitivities to the middle range of spatial frequencies in typical stimulus conditions^5^. In contrast, suprathreshold contrast matching functions exhibit a diminishing spatial frequency dependency as contrast increases; these functions tend to take the shape of a wide band-pass or even become flat at higher contrast levels^5^. Hillis and Brainard^6^ compared color discrimination and color appearance matching in different scenes where luminance changes were created as shadows or paintings. They found that the discrimination sensitivity did not change with different scenes, whereas color appearance, which can be considered as a kind of suprathreshold perception, was affected by scenes. Similarly, Sato, Nagai, Kuriki, and Nakauchi^7^ compared the effects of incomplete adaptation to a reddish background on the sensitivities of color discrimination and on the perception of suprathreshold color differences. Their results indicated that color discrimination was most sensitive to the background color, whereas suprathreshold color difference perception was most sensitive to chromaticity near the perceptually achromatic color. These findings imply that color discrimination and suprathreshold color difference perception might rely on distinct mechanisms with different color representation characteristics.

Categorical perception may partly account for the different characteristics of sensitivities between color discrimination and suprathreshold color difference perception. It has been reported that perceived differences between colors may intensify at category boundaries, leading to a sudden change in perceived color when crossing these boundaries^8,9^. For example, in the visible spectrum, we perceive blue around 450 nm, and it stays blue until around 480 nm, where it shifts to green. It stays green until around 570 nm and then changes to yellow, orange, red, etc.^10^. This phenomenon of perceiving larger color differences around category boundaries is often called “category effects.” The category effects imply that our linguistic categorization of colors influences how we see them^11^. Previous studies found no category effects on the perception of subtle color differences close to discrimination thresholds. For example, Witzel and Gegenfurtner^3^ measured JNDs on an isoluminant hue circle in the Derrington–Krauskopf–Lennie (DKL) color space^12,13^, as well as category boundaries on the same hue circle. They found no correlation between color category boundaries and the JNDs. On the other hand, many previous studies showed category effects on the perception of suprathreshold color differences^14–16^. For example, Roberson et al.^14^ conducted color-matching experiments in which a target stimulus was followed by the simultaneous presentation of two stimuli. The two stimuli were one or two Munsell steps apart (i.e., suprathreshold differences) on the blue/green region, and one of them had the same color as the target. The observers responded which stimulus color matched the target stimulus. Greater accuracy was observed for cross-category judgments than for within-category judgments. In summary, no category effect was observed on color discrimination, the smallest level of color difference, whereas category effects were confirmed on the perception of suprathreshold color differences. These results imply that category effects may depend on the magnitude of color differences.

Based on the above findings, we propose an assumption that as the color difference magnitude increases, people rely more on color categories to judge the color difference; in other words, the category effects on color difference sensitivity become stronger. In the subtle color difference, low-level color signals may be enough to make the judgment. In contrast, when the color difference becomes larger, it may become harder to make judgments only with low-level color signals, and thus, color category information may become more influential on color difference perception.

However, our assumption about the relationship between category effects and the magnitudes of color differences on color sensitivities has not been quantitatively examined. Previous studies have primarily focused on comparing perception between discrimination and some specific scales of suprathreshold color difference^7^. In addition, category effects were only confirmed on some specific magnitude of color difference perception. For instance, Witzel and Gegenfurtner^15^ employed 2-JND stimuli to investigate the characteristics of suprathreshold perception. Furthermore, earlier studies on categorical color perception focused on only some specific color regions, such as the blue/green boundary in the Munsell space^11,16^. How the perceptual strategy changes in entire color hues depending on the magnitude of color differences, from subtle ones near the discrimination thresholds to large ones above the discrimination thresholds, is still largely unknown.

This study aims to examine the sensitivity transition across various magnitudes of color difference and uncover the underlying mechanisms. We measure the sensitivities of color discrimination and the perception of two sizes of suprathreshold color differences on the same stimuli colors. First, we quantitatively compare sensitivity characteristics across different hues among discrimination, perception of small color difference, and perception of large color differences. Then, we analyze the relationship between the sensitivity characteristics and color categories, which are measured in a separate experiment.

## Results

### Color discrimination

First, we measured hue discrimination thresholds at 32 pedestal colors. These pedestal colors were uniformly selected from a hue circle whose center was the chromaticity of D65 illuminant on the CIE1976 *u'v'* chromaticity diagram. We employed the PSI adaptive staircase method^17^ to measure discrimination thresholds. Individual observers’ thresholds were estimated by fitting logistic functions to correct response ratios as a function of stimulus hue differences. Discrimination sensitivity was then defined as the reciprocal of these thresholds. Then, we normalized the sensitivity at each pedestal color by dividing it by the mean sensitivity across 32 pedestal colors in each observer’s results. Finally, we averaged the normalized sensitivity across the observers.

**Figure 1:**
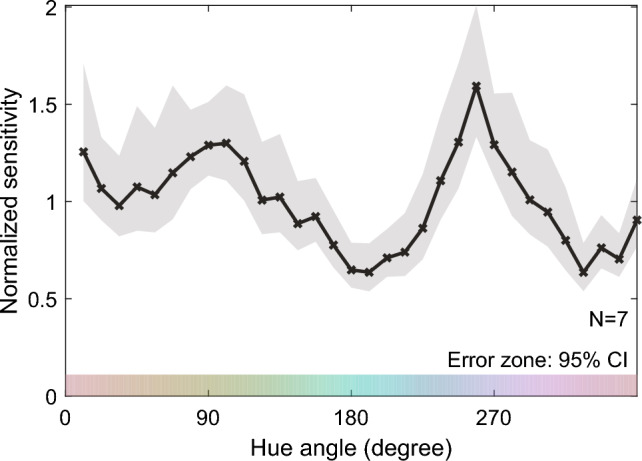
Normalized color discrimination sensitivity averaged across all observers. The horizontal axis represents the hue angle of the pedestal color, and the vertical axis represents the normalized discrimination sensitivity. The error zone (gray shaded area) indicates the 95% confidence intervals, which were computed through a parametric bootstrap procedure with 10,000 iterations.

Figure 1 shows the normalized discrimination sensitivities averaged across observers as a function of the pedestal color. The sensitivities differed across the hue angles, even though the pedestal colors were defined on the uniform chromaticity diagram. Notably, there were clear sensitivity peaks around 250° and 90°.

### Suprathreshold color difference

Then, we measured the sensitivities to suprathreshold color differences. We employed the Maximum Likelihood Difference Scaling (MLDS) method^18^, which is used to measure perceptual scales (interval scales) for several levels of stimuli based on the observer’s responses. In a trial, a triplet of squares with different colors was presented. The three squares (A, B, and C) constituted two pairs: the left pair (A, B) and the right pair (B, C), and their colors were selected from the 32 sample colors, which were the same as the pedestal color in the discrimination experiment. The observer judged which pair had a larger color difference. MLDS assumes that a common mechanism represents the perceptual scale regardless of the physical differences in stimuli. However, this study explores the possibility that color representations for perceiving color differences might vary with the sizes of color differences, which contradicts the MLDS assumption. Thus, we first investigated whether the observer responses in the MLDS method exhibited different properties between the small and large color differences by analyzing the responses for the two sizes of color differences separately.

**Figure 2:**
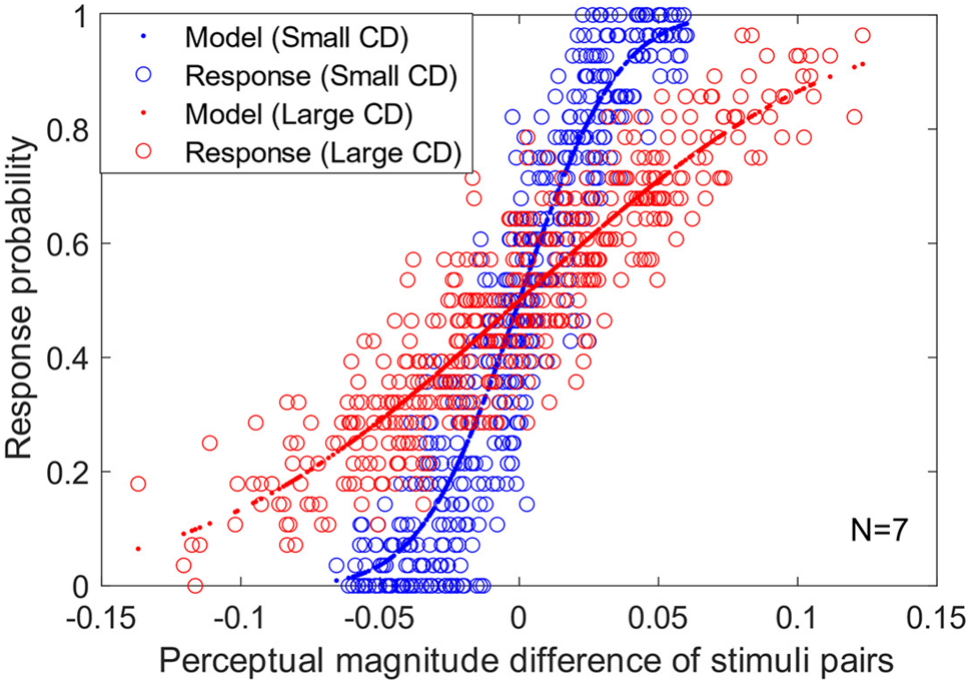
Response probabilities for small and large color differences in MLDS experiment. The horizontal axis represents the perceptual color difference between the left and right pairs in the MLDS model, which was estimated in MLDS model fitting. The vertical axis represents the probability of the response “left stimulus pair had larger color difference.” The data points marked with “o” depict the actual response probabilities from the observers, whereas the data points marked with “.” represent the response probabilities derived from the response model in MLDS. The small color differences are denoted by blue dots, while the large color differences are denoted by the red dots.

In MLDS, the perceptual scales for different stimuli were adjusted so that response probabilities matched the prediction by the response model. Figure 2 illustrates the response probabilities for the small and large color differences to compare the response variability, because the variance of the color judgments has often been used as an index to discover the properties of the mechanism underlying color perception^19^. The horizontal axis shows differences in perceptual color differences between two stimulus pairs estimated by the MLDS model; the larger value means the left pair had larger perceptual color differences within the two pairs. The vertical axis shows the probability of the left pair selection. The two psychometric functions (logistic functions) represented with small dots “.”, which were fitted to the observer responses, had different slopes; the large color differences (the red line) had a shallower slope. This suggests that internal noise for the perception of large color differences was larger than that for small color differences. Indeed, the estimated noise in the MLDS model (described in Methods in more detail) was much larger for the large color difference (0.09 and 0.03 for the large and small color differences, respectively). This slope difference does not rule out the possibility that the mechanisms underlying the perception of the two sizes of color differences may have different characteristics. This possibility is important in this study since we are interested in the transition of mechanisms depending on the sizes of the color differences. Hence, we determined to analyze them separately.

**Figure 3:**
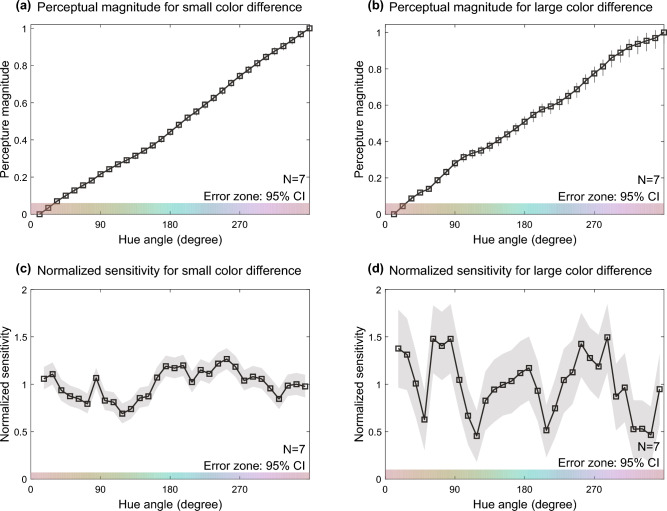
Results of suprathreshold color difference experiment. (**a**) The perceptual scale for small color differences. (**b**) The perceptual scale for large color differences. (**c**) The normalized sensitivity for small color differences. (**d**) The normalized sensitivity for large color differences. The horizontal axis represents the hue angle. The vertical axis represents the perceptual scale or normalized sensitivity. The error zones are the 95% confidence intervals obtained from the parametric bootstrap procedure with 10,000 repetitions. These perceptual scales and sensitivities were computed from responses made by seven observers.

Figure 3 shows the results of the MLDS procedure. Figure 3a,b illustrate the perceptual scales for the small and large color differences, respectively. The perceptual scale of small color differences in Fig. 3a had a smoother trend than that of large color differences in Fig. 3b. Figure 3c,d illustrate the normalized sensitivities. They were calculated in two steps; first, the sensitivities were calculated as the difference in perceptual scales between adjacent sample colors, and then the normalized sensitivities were calculated by dividing the sensitivities by their mean across hues. The normalized sensitivity tends to be more peak-like for large color differences in Fig. 3d. Notably, unlike the color discrimination sensitivities, which had only two main peaks, the sensitivities to suprathreshold color differences had multiple sensitivity peaks for both small and large color differences.

### Comparison between color discrimination and suprathreshold color difference perception

**Figure 4:**
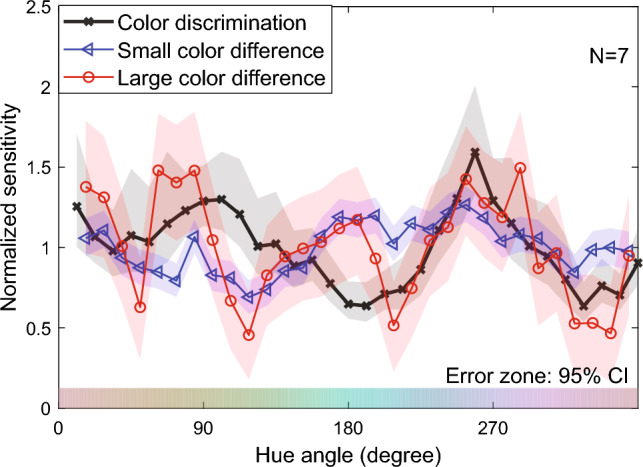
Normalized sensitivities of color discrimination, small suprathreshold color differences, and large suprathreshold color differences. The horizontal axis represents the hue angle, and the vertical axis represents the normalized sensitivity. The error zone (shaded area) indicates the 95% confidence intervals, which were computed through a parametric bootstrap procedure involving 10,000 iterations.

The normalized sensitivities for color discrimination, small suprathreshold color difference perception, and large suprathreshold color difference perception can be compared between the three types of color differences. Figure 4 shows the three types of normalized sensitivities in a panel. The sensitivity characteristics differed significantly between color discrimination and the suprathreshold color differences. For example, color discrimination and suprathreshold color difference perception displayed roughly opposite trends within the hue angle range from 90° to 270°. This clear difference is consistent with the hypothesis posited by Sato et al.^7^ that color discrimination and the perception of suprathreshold color differences involve distinct mechanisms. Moreover, even the sensitivities of two sizes of suprathreshold color differences showed different trends; the perception of large color differences exhibited larger amplitudes and a more pronounced peak-like pattern in their sensitivity profiles. These results indicate that the sensitivity characteristics differ between color discrimination and suprathreshold color difference perception and even within the perceptions of suprathreshold color differences.

### Relationship with color category boundary

We demonstrated that the sensitivity characteristics varied with the color difference sizes. Thus, we investigated the category effects on the color difference sensitivities to test our assumption that the category effects on color difference sensitivity increase with the color difference size. First, we conducted an additional experiment to measure the category boundaries. We then plotted the category boundaries on the sensitivity chart of color difference perception to explore their relationship.

However, comparing the sensitivities and the color category boundaries might be problematic. We chose the same 32 colors as the pedestal colors for the discrimination experiment and the sample colors for the suprathreshold color difference experiment. In addition, to achieve perceptual uniformity of color differences, these colors were uniformly chosen along a hue circle on the *u'v'* chromaticity diagram. However, the Euclidean distance on the * u'v'* diagram does not necessarily reflect the perceptual color difference; that is, the nonuniformity of the *u'v'* diagram could also affect the sensitivity profiles. In addition, we were mainly interested in the differences in the sensitivity profiles among the color difference sizes. To address this issue, we normalized the sensitivities for suprathreshold color differences by dividing them by the corresponding color discrimination thresholds. We refer to these normalized values as the relative sensitivities.

**Figure 5:**
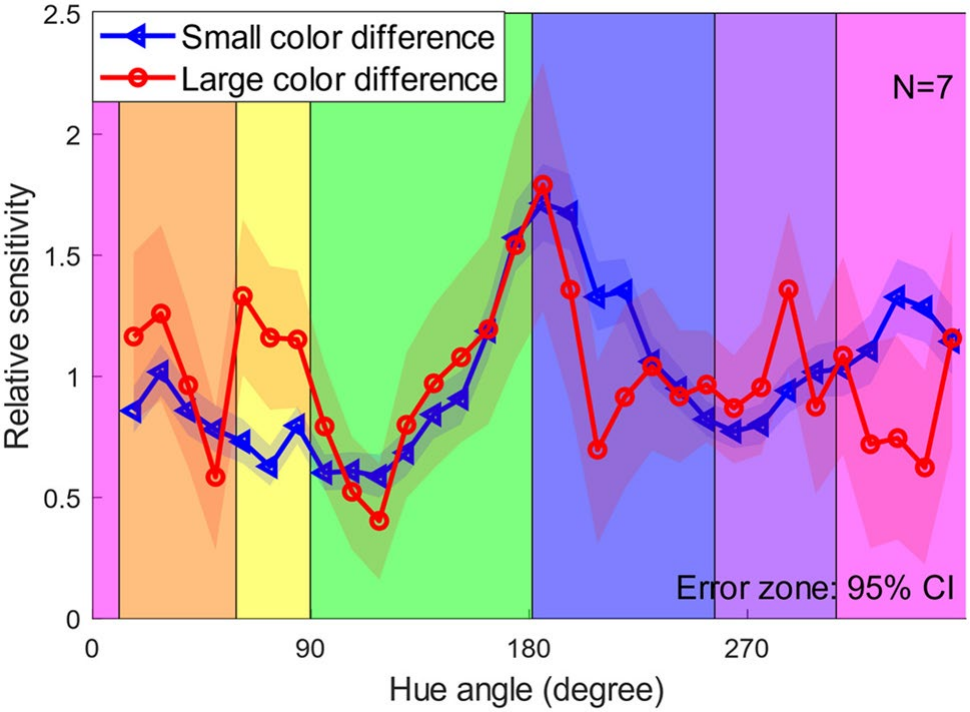
Relative sensitivities for small and large suprathreshold color difference. The horizontal axis represents the hue angle, and the vertical axis represents the relative sensitivities (see text for details). The blue and red lines represent the small and large color differences, respectively. The vertical black lines represent the category boundaries measured in an additional experiment.

Figure 5 shows the relative sensitivities for the small and large color differences. Sensitivity peaks were observed at four out of six category boundaries: pink/orange, orange/yellow, yellow/green, and green/blue, especially for the large color difference. This may suggest the existence of category effects on the perception of suprathreshold color differences. In contrast, relatively weaker category effects were found for the small color differences. This implies that categorical effects were more prominent for the larger color differences. However, not all the category boundaries exhibited sensitivity peaks; for instance, the blue/purple (around 256°) boundary did not exhibit a sensitivity peak. The reason may be that a peak was already present in the sensitivity profiles for both color discrimination and suprathreshold color difference (as shown in Fig. 4), and thus the peak was diminished by the normalization process.

We used a two-tailed parametric bootstrap test with 10,000 iterations and a significance level of 5% to test the differences in relative sensitivities between every hue angle pair. Given the multiple comparisons in this test, we applied the Holm method for significance level correction. We found significant differences in small color difference sensitivities between some hue angle pairs (90 of 465 pairs, *p* < 0.05 after Holm correction). Those in large color difference sensitivities were also found in some hue angle pairs (20 of 465 pairs, *p* < 0.05). In addition, differences in relative sensitivities between the small and large color differences at each hue angle were also tested with the bootstrap procedure in a similar way. The results showed that the differences were statistically significant for two of 31 hue angles (*p* < 0.05).

### Model analysis of color difference sensitivity

**Figure 6:**
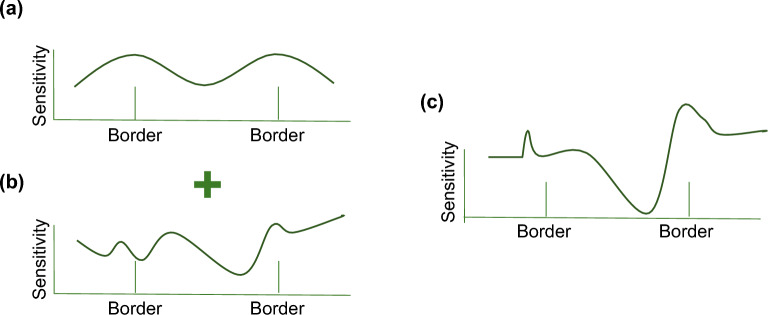
Concept of model analysis to represent relative color difference sensitivity. (**a**) Gaussian models that describe category effects. (**b**) Munsell color difference that forms the baseline sensitivity. (**c**) Our model sensitivity represented as the sum of (**a**) and (**b**).

The results above qualitatively suggest the relationship between the color category and the suprathreshold color difference sensitivity, especially for the large color differences. To examine this relationship more quantitatively, we fitted a simple model to our experimental data. Figure 6 illustrates the concept of the model; it assumed that color difference perception was determined by both color category effects and some baseline mechanisms. Therefore, our model (Fig. 6c) represented the relative color differences by the sum of category effects (Fig. 6a) and the baseline (Fig. 6b).

The category effects in our model were described by Gaussian function as shown in Eq. (1):

1 $$ f_{c} \left( {x, \;w_{c} ,\;\mu_{c} , \;\sigma } \right) = w_{c} \exp \left\{ { - \frac{{(x - \mu_{c} )^{2} }}{{2\sigma^{2} }}} \right\}, \quad \left( {c = 1,\;2, \ldots 6} \right) $$

where $c$ is the index for each category boundary (pink/orange, orange/yellow, yellow/green, green/blue, blue/purple, or purple/pink), $\mu_{c}$ $\left( {c = 1,\;2, \ldots 6} \right)$ is the hue angle of each color category boundary, $\sigma$ is the standard deviation shared by all categories, and *w*_c_ $\left( {c = 1,\;2,\; \ldots 6} \right)$ is the weight of each categorical boundary to the color difference sensitivities.

Then, we used Munsell color space to represent the baseline of the suprathreshold color differences, because the Munsell space is considered to better represent color appearance than the *u'v'* chromaticity diagram, which is not necessarily suitable for representing perceptual color appearance as shown in the Abney effect^20^. The baseline component is shown in Eq. (2):

2 $$ f_{m} \left( {x, \;w_{m} } \right) = w_{m} M, $$

where $w_{m}$ is the weight of Munsell space on color difference sensitivity, and $M$ is the Euclidean distance of the adjacent colors of our 32 colors in the Munsell space. The details are described as follows:

1. Estimate the color discrimination sensitivities in the Munsell space.
  ① Convert the discrimination threshold at each of the 32 pedestal colors in the *u'v'* chromaticity diagram to the Euclidean difference in Munsell space. This value is called the “Munsell threshold.”
  ② Calculate the sensitivity as the reciprocal of the Munsell threshold.
  ③ Normalize the sensitivities by their mean across the 32 pedestal colors.
2. Estimate the sensitivities to the suprathreshold color differences based on Munsell space.
  ① Convert the 32 sample colors in the *u'v'* chromaticity diagram to the Munsell space.
  ② Calculate the Euclidean differences between the adjacent colors in the Munsell space. This value was referred to as Munsell sensitivity.
  ③ Normalize all Munsell sensitivities by their mean across the 32 sample colors.
3. Normalized the sensitivities in (2) by dividing them by the discrimination sensitivities in (1)

Please note that *M* is independent of the color difference size (small or large).

Finally, we added these two components based on color categories and the Munsell space to represent the perceptual color differences measured in the experiment:

3 $$ f_{total} = \mathop \sum \limits_{1}^{6} f_{c} \left( {x,\;w_{c} ,\;\mu_{c} ,\;\sigma } \right) + f_{m} \left( {x,\;w_{m} } \right) + C, $$

where $C$ is the constant term.

**Figure 7:**
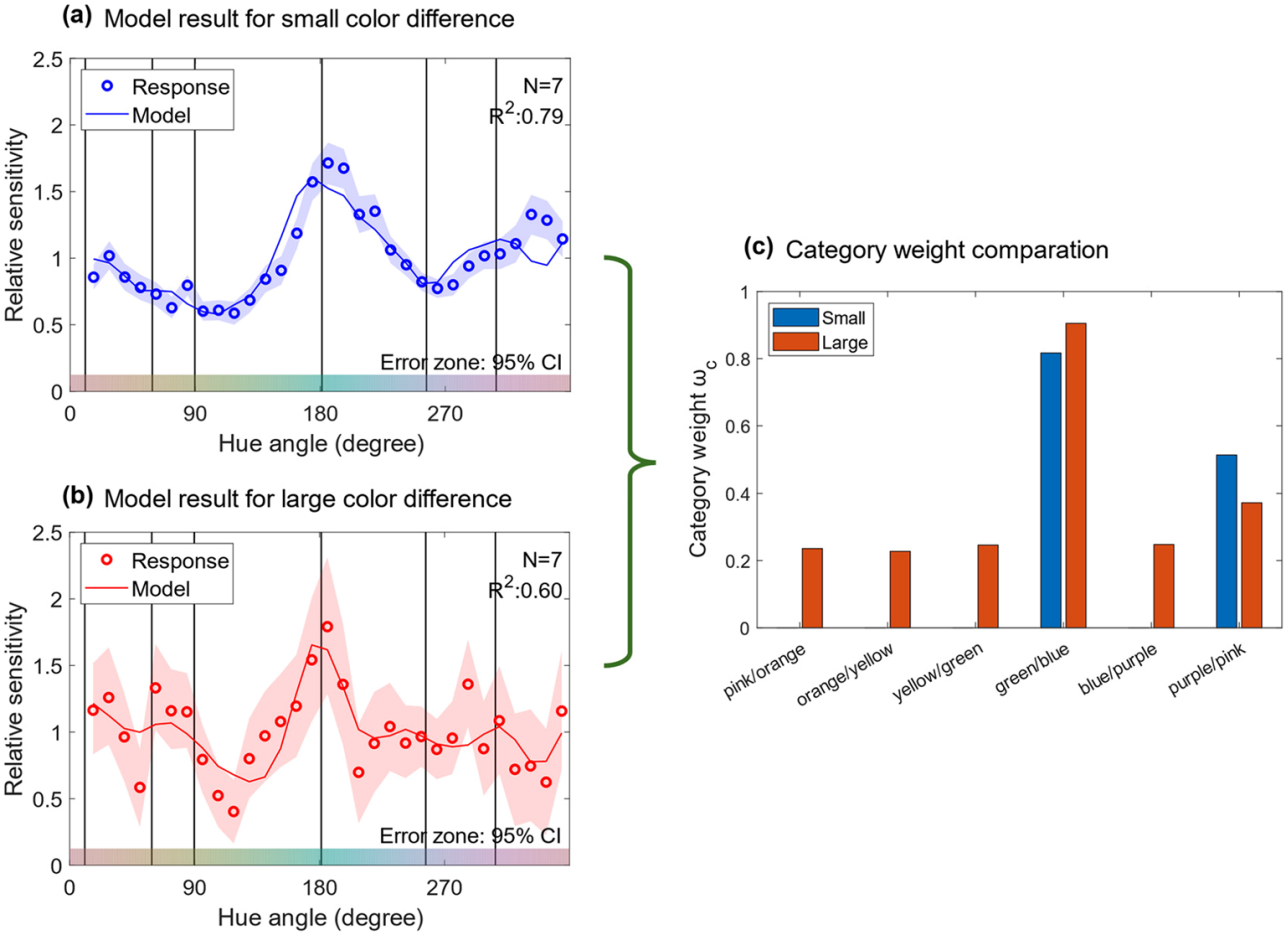
Results of model fitting to relative sensitivity for (**a**) small color differences and (**b**) large color differences. Circles represent the relative sensitivities measured in the experiment, and the solid line represents the model output. (**c**) Category weights of six category boundaries ($w_{c}$ ($c = 1,\;2, \ldots 6$)) in the model for small and large color differences. The blue and red bars show the category weights for the small and large color differences, respectively.

This model was fitted to each of the relative sensitivities of small and large color differences using a nonlinear least-squares method; here, we used the *lsqcurvefit* function in MATLAB for this purpose. Since $$\mu_{c} \left( {c = 1,\;2, \ldots 6} \right)$$ were measured in the category experiment, the free parameters of this model were $$w_{c} \left( {c = 1,\;2, \ldots 6} \right)$$, $$w_{m}$$, $$\sigma$$ and *C*. Figure 7a,b illustrate the response data and fitted model for the small and large color differences, respectively. Figure 7c shows the category weights $$w_{c} \left( {c = 1,\;2, \ldots 6} \right)$$ for the small and large color differences. In the small color difference, $$w_{c} $$ were greater than 0 only for the green/blue and purple/pink boundaries, while in the large color difference, $$w_{c}$$ were greater than 0 for all category boundaries. These results suggest that color category effects are limited to the two boundaries for small color difference perception but are present at all boundaries for large color difference perception. In addition, the average category weight across the category boundaries was significantly higher for the large color difference than for the small color difference based on the two-tailed parametric bootstrap test with 10,000 iterations (*p* < 0.01).

## Discussion

We measured the sensitivities along the hues for three sizes of color difference: color discrimination, suprathreshold small color differences, and suprathreshold large color differences. We found that the sensitivity profiles as a function of stimulus hue varied among these three sizes. This variation is consistent with the idea that different mechanisms underlie the perception of the different sizes of color differences^7^. Furthermore, we found that color difference sensitivities, especially for the large color differences, exhibited peaks at four of the six color category boundaries. We quantitatively analyzed the relationship between the sensitivity profiles for color difference and color categories based on the simple model. The model revealed that the weights of category boundaries on the color difference sensitivities were higher for the large color differences than for the small color differences, suggesting that the category effects became more obvious as the color difference increased. These findings suggest that the color categories contribute to the judgments of color differences to some extent, especially when judging large color differences.

The difference in the weights of color category boundaries between the small and large color differences may arise from the reliability of bottom-up color signals. The judgment or perception of color differences typically becomes difficult with their sizes, as shown in the shallow slope (i.e., larger noise) of the MLDS psychometric functions in Fig. 2. This difficulty is obvious from daily experiences: for instance, although we can easily discriminate the colors of blue and red books, quantitative evaluation of the color difference is severely difficult. In this situation, the reliability of quantitative color signals for perceptual judgments may be very low. Therefore, our visual system may rely more on categorical color perception, which is more discrete but stable than quantitative color signals, for the color difference perception. This speculation may be tested by adjusting the reliability of bottom-up color signals, for example, by adding spatiotemporal noise to the stimuli.

We did not find category effects at all the category boundaries; rather, we observed them only at four of the six boundaries, excluding the blue/purple and purple/pink boundaries. The following are some possible reasons for the lack of category effects at the two boundaries.

**Figure 8:**
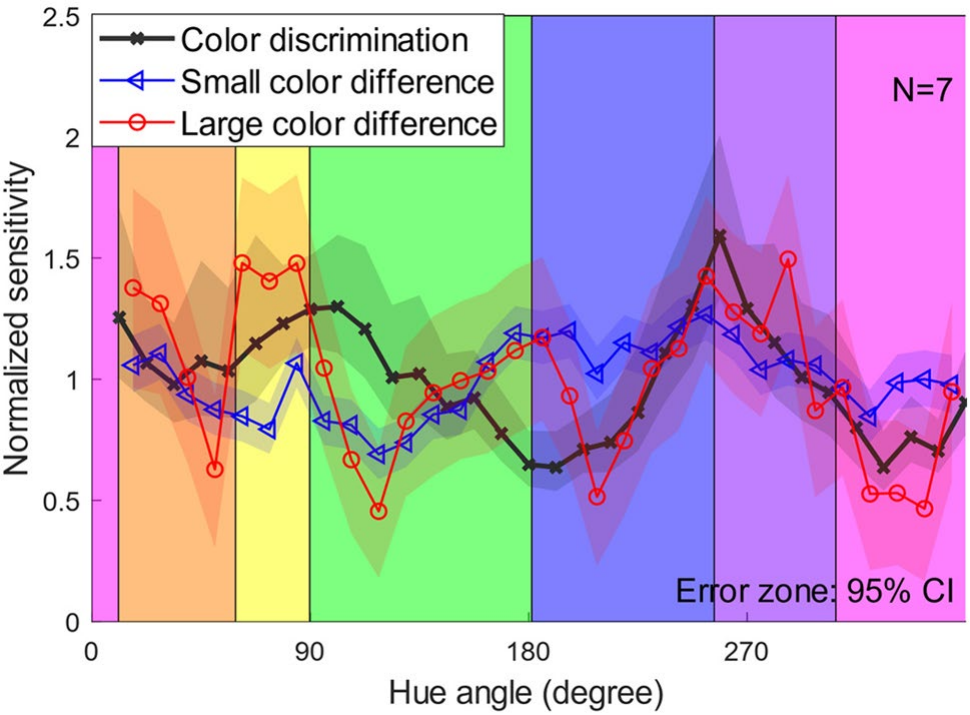
Normalized sensitivity of three levels of color differences superimposed on category boundaries. The line charts are the same as Fig. 4.

Two possible interpretations exist for the lack of effect on the blue/purple category boundary. The first one is that the blue/purple boundary has no impact on color difference perception. This is supported by our results, which showed that the sensitivity peak at the boundary in the large color difference perception vanished after compensation based on the color discrimination sensitivity. The compensation effects can be found by comparing Fig. 8, which illustrates the sensitivities before compensation (the same data as in Fig. 4) superimposed on the category boundaries, and Fig. 5. This suggests the possibility that this peak was an artifact of the color space distortion. A previous study on color working memory^21^ is compatible with this idea. This study performed an experiment where the observer reproduced colors of color stimuli based on their short-term memory and found that the reproduced colors tended to be biased toward the color category centers. Furthermore, a quantitative model suggested that this bias was absent for the purple category. This is consistent with our results. This study also revealed that the responses for the purple category overlapped considerably with those for the adjacent blue and pink categories. The purple category’s lack of distinctiveness might have been related to its ineffectiveness. The other is that the blue/purple category did affect color difference perception, but its effect was masked by the sensitivity compensation. As we mentioned above, larger color difference of stimuli make the color difference judgment more difficult. This implies that in MLDS, the observer’s responses become more variable, and the hue sensitivity profile becomes flatter. Therefore, one could argue that the blue/purple boundary additionally improved the color difference sensitivity from the flatter sensitivity profile. Indeed, the improvement of reaction times at the blue/purple boundary has been reported^22^. However, these two possibilities are indistinguishable from our results. Future experiments should test these two interpretations by altering the color space, which would allow us to control the presence or absence of the blue-purple peak in the discrimination sensitivity profile.

On the other hand, there was no sensitivity peak around the purple/pink boundary in any of the color difference sizes, as shown in Fig. 8. Thus, the conclusion that the boundary does not have any effects on color difference perception seems plausible. Although the origin of color categories has been debated for many decades, some have argued that color lexicons are biologically constrained in their evolution^23–25^. For example, Skelton et al.^25^ provided evidence that even infants have color categories for red, yellow, green, blue, and purple. On the other hand, others have argued that color terms and their categories are culturally and linguistically constructed^14,26^. As a result, it is generally accepted that color categories are neither entirely biological nor entirely cultural and linguistic in origin^26^. This idea raises the possibility that different color categories influence perception in different ways. For instance, the fact that pink is not included in infants’ categories^25^ may be related to the absence of category effects at the pink/purple boundary.

The mechanisms underlying the category effect on color difference sensitivity are still unclear. One possible hypothesis is that color difference perception becomes more dependent on the category as the color difference increases. In contrast, Witzel and Gegenfurtner suggested that category effects were due to the observers focusing their attention on the differences between linguistic categories, which is called “categorical facilitation”^15^. In their study, they measured response times and error rates in a speeded color discrimination task involving stimuli with 2-JND color differences. The results showed that the response times and error rates were lowest for stimulus pairs around category boundaries and highest for pairs within a single category. Interestingly, this pattern was evident among inexperienced observers who had not experienced JND measurement but not among trained observers who were highly familiar with JND measurement. The inexperienced observers may have paid attention to and relied on linguistic distinctions due to their unfamiliarity with judgments of small sensory signal differences. On the other hand, the trained group might not need to rely heavily on linguistic distinctions because of their familiarity with using small sensory signal differences. In contrast, our observers were not completely inexperienced, as they had experienced the color discrimination experiment before the suprathreshold color difference experiments. However, because our color differences were much larger than those in Witzel et al.^15^, where bottom-up signals were less reliable, a similar explanation as categorical facilitation might apply to our results, even for well-trained individuals. Of course, we do not have clear evidence of whether the sensitivity peaks around the category boundaries resulted from an intentional change in attention to linguistic categories or not. Our study did not explicitly control the degree of attention to linguistic categories, nor did Witzel and Gegenfurtner^15^. We leave these questions for future studies.

The effects of color categories were not supported in some previous studies. For example, Smallman and Boynton^27^ found that colors can be efficiently segregated based on their separation in color space, irrespective of their colors’ basic color categories. In contrast, our results suggest that category boundaries between basic categories increase color difference sensitivities, particularly for stimuli with larger color differences. The apparent discrepancy in claims may stem from differences in the experimental designs. Our study systematically investigated the characteristics of color difference perception across various color difference sizes and observed the impacts of the basic categories. However, this does not imply that color difference perception relies solely on category information; rather, both the magnitude of the color difference itself and the categories are considered to play roles. Smallman and Boynton did not systematically control for the magnitude of color differences, making it challenging to quantitatively assess the contributions of color differences and categories. By systematically varying color difference sizes, we can strive for a unified explanation of color segregation and color difference perception, considering both the magnitude of color differences and categorical distinctions.

The sensitivity profile itself along the hue is challenging to interpret. In this study, we eliminated the effects of the baseline sensitivity profiles, such as the color space distortion, by comparing the sensitivities between different sizes of color differences. However, the sensitivity profile along the hue superficially appears to deviate from the previous studies. For instance, the sensitivity inferred from the variability of the unique white setting was low along the blue-yellow direction^28^. This seems rather inconsistent with our finding of higher sensitivity around 270 degrees, purplish or blueish colors. However, the previous studies assessed the discrimination sensitivities near the adaptation color, whereas we measured it at the pedestals distant from the adaptation color, and these two kinds of discrimination are known to exhibit distinct properties^29^. Thus, the discrepancy between the studies is not unexpected, and a comprehensive account of these results requires an understanding of the mechanisms underlying the shift in the color discrimination sensitivities from the adaptation color to the pedestal. The stimulus size should also affect the sensitivity profiles across hues. All our experimental stimuli were sized at 2° in visual angle, which corresponds to the standard observer’s visual subtense in colorimetry. Smaller stimuli would decrease discrimination sensitivities for blueish and greenish colors—a phenomenon known as small-field tritanopia^30^. Conversely, larger stimuli might make bluish colors more vivid. Therefore, the sensitivity profiles (and possibly the relative sensitivities) measured in this study might apply only to the visual subtense we employed. Future studies should investigate the effects of stimulus sizes.

Several important questions remain unanswered. The first issue is to explore the relationship between attention and category effects, as stated above. An experiment that controls attention to linguistic information may help test the “categorical facilitation” hypothesis. The second issue is to try alternative experimental methods for measuring perceptual color differences. The sensitivity profiles of suprathreshold color difference perception in this study may have had potential distortions, since MLDS assumes that a common mechanism represents the perceptual scale, the validity of which is doubtful. Measuring perceptual color differences using alternative methods, such as cross-feature matching to luminance difference, may help validate the robustness of our sensitivity profiles. The last issue is to vary stimulus luminance. We used fixed luminance stimuli of 20 cd/m^2^ in our experiment. However, luminance is known to change the color category boundaries. The relations between the sensitivity profile and category boundaries can be explored by employing different luminance conditions.

## Methods

### General methods

The same apparatus and observers were employed for all the experiments: color discrimination, suprathreshold color difference, and color category. We selected stimulus colors with approximately equal saturation along an isoluminant hue circle in *u'v'* chromaticity diagram at a fixed luminance of 20 cd/m^2^. In this chromaticity diagram, the 270 degrees correspond to purplish color. The isoluminance of every individual observer was measured with heterochromatic flicker photometry before the main experiments. In the first experiment, we measured color discrimination thresholds using the PSI adaptive staircase procedure. In the second experiment, we measured the sensitivities to different magnitudes of suprathreshold color differences using the MLDS method. Finally, we measured color category boundaries in a categorical color naming experiment.

### Observers

The observers were two females and five males. All were undergraduate or graduate students from Tokyo Institute of Technology. Three observers were native speakers of Japanese, and four were native speakers of Chinese. All observers passed the Ishihara color vision test and had normal or corrected-to-normal visual acuity.

### Ethical approval and informed consent

All experiments were designed in accordance with the Declaration of Helsinki and was approved by the Ethical Review Committee of Tokyo Institute of Technology. Informed written consent was obtained from all observers after explaining the details of experimental protocols.

### Apparatus

The experiments were conducted in a darkroom. The stimuli were presented on an LCD monitor (EV2785, EIZO, Japan) with a color resolution of 8 bits per channel, a spatial resolution of 3840 × 2160 pixels, and a refresh rate of 60 Hz. The spectra and gamma properties of the monitor primaries were carefully measured with a spectroradiometer (Specbos1211-2, JETI Technische Instrumente GmbH, Germany) and a colorimeter (ColorCAL II, Cambridge Research Systems, UK), respectively. Based on these measurements, color look-up tables were created to accurately present the desired luminance and chromaticity. The monitor was connected to a desktop computer (HP Japan; CPU Core i7-9700F, GPU NVIDIA GeForce RTX 2060, OS Ubuntu 20.04LTS). Experiments were controlled by custom programs written in MATLAB R2021a (The MathWorks Inc., USA) with Psychtoolbox3^31–33^ and Palamedes Toolbox^34^ extensions. The observer’s head was approximately fixed by a chinrest at a viewing distance of 66 cm. The observer saw the stimuli with binocular natural vision. They used a numeric keypad for the responses.

The LCD monitor has a limited color resolution of 8 bits, which is often insufficient to capture the color discrimination thresholds. To overcome this limitation, we applied the Noisy-Bit Method (NBM)^35^, which is a software technique that enables virtually infinite color resolutions. In NBM, two digital values close to the desired pixel intensity is alternately and randomly presented; more specifically, a spatiotemporal noise is presented whose probability summation matches the desired intensity. For example, if the desired R-value is 50.25, the values 50 and 51 are presented with 75% and 25% probabilities, respectively, as a spatiotemporal noise.

### Color space

**Figure 9:**
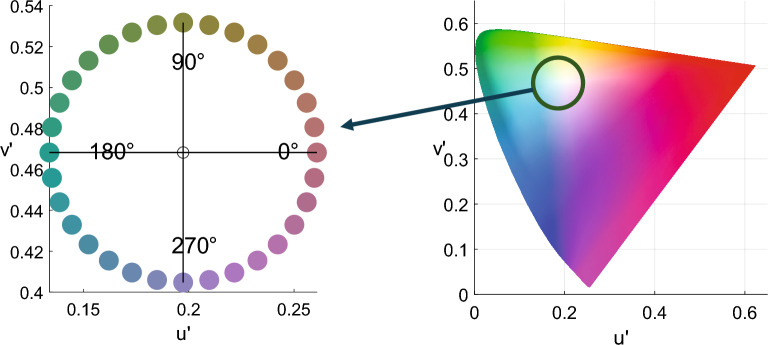
Stimulus colors on the CIE 1976 *u'v'* chromaticity diagram. We sampled them from this hue circle and represented them by their hue angle. For instance, the color on the positive *u'* axis was written as “0°” color.

The stimulus colors were defined in the CIE 1976 *u'v'* chromaticity diagram. The luminance of the stimuli was fixed at 20 cd/m^2^. Except for the heterochromatic flicker photometry (HFP) experiment, the stimulus colors were selected from a hue circle shown in Fig. 9. The center of the hue circle was the chromaticity of illuminant D65. The saturation (the radius of the circle) was determined so that the saturation was maximum within the monitor color gamut. In contrast, for the HFP experiment, we used the so-called Derrington–Krauskopf–Lennie (DKL) color space based on the cone fundamentals proposed by Stockman and Sharpe 2000^36^. The L–M and S axes were normalized by the monitor color gamut to [− 1,1], and the origin was set to the chromaticity of D65 with 20 cd/m^2^.

### Isoluminance measurement with heterochromatic flicker photometry

This study aimed to measure sensitivities to different sizes of color differences. However, luminance differences in the stimuli can affect color difference sensitivity as an artifact, specifically color discrimination sensitivity, even if the luminance differences are subtle. To eliminate this confounding factor, we measured the subjective isoluminance for each observer using HFP and adjusted experimental stimuli to be isoluminant accordingly.

### Stimulus

**Figure 10:**
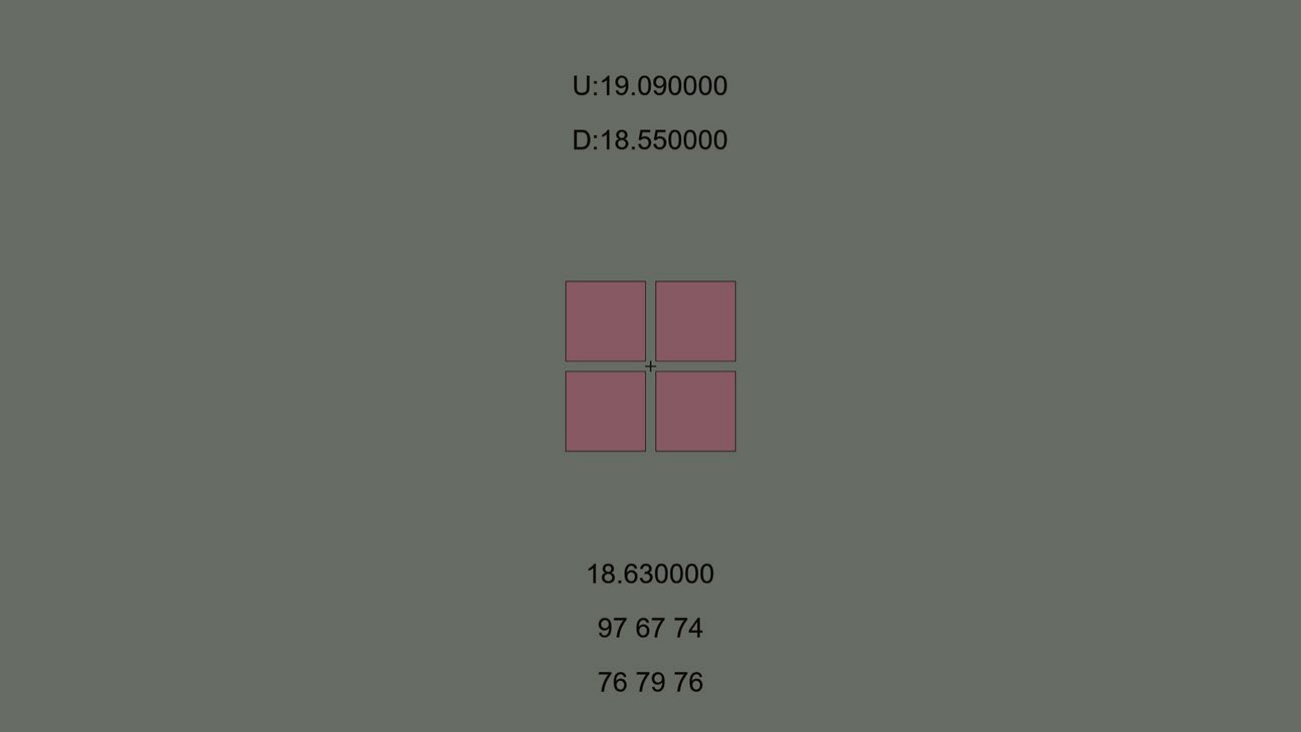
Stimulus example in HFP experiment. Four squares with the same color were displayed in the screen center. The numbers were debugging information that was forgotten to be removed: “U:19.090000” and “D: 18.55000” represent the upper and lower thresholds (cd/m^2^) set by the observer, “18.630000” represented the current luminance of the stimuli, “97 67 74” represents the RGB DAC values of the stimuli, and “76 79 76” represents the RGB DAC values of the background. Although the values were visible to the observers, they were not informed of the meanings of the values.

Figure 10 illustrates an example of the stimulus. It was composed of four squares presented on a gray background of the origin color. They had the same chromatic color, each with 1-pixel black edges. Each square’s width and height were 2° in visual angle, and the interval between the squares was 0.25°. During the stimulus presentation, the square colors of the four squares temporally alternated between the gray background (the center color of the DKL color space, 20 cd/m^2^) and a chromatic color at a frequency of 20 Hz. The chromatic color was sampled from the L–M axis, with 12 equally spaced values ranging from − 0.99 to 0.99, while the S − (L + M) value was fixed at 0. The luminance of the chromatic color was adjusted by the observer. The initial luminance of the chromatic color was randomly chosen between 15 and 25 cd/m^2^.

### Procedure

In each trial, a chromatic color in the squares was randomly selected from the 12 colors. The observer was asked to find both the upper and lower luminance thresholds of the minimum flickering zone by decreasing (using the keypad’s “8” key) or increasing (using the keypad’s “2” key) the luminance of the chromatic color. There was no time limitation during the observer’s adjusting. When the observer found the thresholds, they pressed corresponding keys to record the threshold luminance (“+” key and “−” key for upper and lower thresholds, respectively); that is, two responses were requested in each trial. After completing the responses for both thresholds, the observer pressed a confirmation key to proceed to the next trial. Each chromatic color was tested only once. We conducted one session with 12 trials.

### Analysis

We first averaged the lower and upper thresholds for each color. Then, we conducted a linear regression analysis between the averaged threshold (isoluminant point) and the L–M value for each observer. We utilized the slope of the regression line to make different colors isoluminant in the color discrimination experiment.

### Color discrimination experiment

To quantify the sensitivity to the smallest color difference, we measured color discrimination thresholds on 32 pedestal colors with various hues using the PSI adaptive staircase method.

### Stimulus

**Figure 11:**
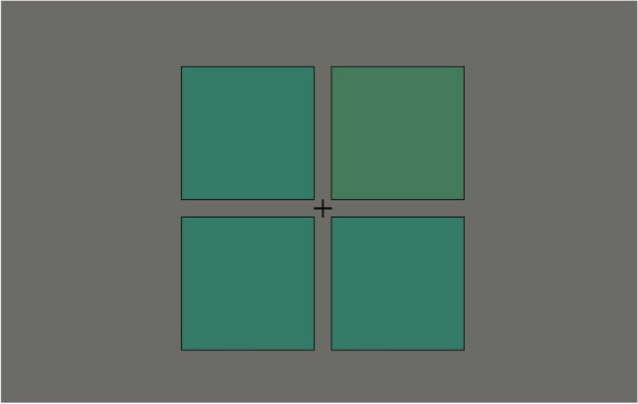
Stimulus in color discrimination experiment.

Figure 11 shows an example of the stimulus. The spatial layout of the stimulus was the same as in the HFP experiment, except that no characters were presented. Three of the four squares were reference squares, and the other was the test square. The reference squares had the same color, but the test square had a slightly different color. The mean hue between the reference and test square colors was chosen from the 32 colors with an equal hue angle interval (11.25°) along the hue circle in the CIE 1976 *u'v'* chromaticity diagram shown in Fig. 9. This hue was referred to as the pedestal color. The test and reference square colors slightly shifted in a counterclockwise and clockwise direction on the hue circle from the pedestal color, respectively. The amount of the color shift, which was the same for the test and reference squares, was determined using the PSI adaptive staircase method based on the observer’s response history. All colors were set individually isoluminant with the gray (hue circle center) of 20 cd/m^2^ based on the results of the HFP experiment. The test square position was chosen randomly from the four positions in each trial.

### Procedure

The squares with a randomly selected pedestal color were presented for 500 ms. The observer had to identify the test square by pressing one of four keys that corresponded to the four positions of the squares in a four-alternative forced-choice (4AFC) manner. A feedback sound was played if the answer was correct, but no sound if the answer was wrong. The subsequent trial started 1000 ms after the observer’s response.

The number of trials for each pedestal color was 200, resulting in a total of 6400 trials (= 32 pedestal colors × 200 trials). These trials were conducted in five sessions, each with 1280 trials (= 32 pedestal colors × 40 trials). The trials for all 32 pedestal colors were included in each session, and their presentation order was randomly determined. The response history of the observer in earlier sessions for the PSI staircase was carried over to the following sessions. Each session lasted approximately 30 min, and the observer took a short break between the sessions. At the beginning of each session, 60 practice trials were conducted using randomly chosen pedestal colors. The practice trials helped the observer become accustomed to the experimental task and adapt to the experimental environment.

### Analysis

Discrimination thresholds were estimated by fitting a logistic function to the observer’s response using a maximum likelihood criterion. The Palamedes Toolbox was used to fit the psychometric function. The 95% confidence intervals were computed with a bootstrapping procedure with 10,000 repetitions. The sensitivity was calculated as the reciprocal of the mean threshold across all observers. The sensitivities were then normalized by dividing them by the mean sensitivity across all pedestal colors. This normalization allowed us to compare them with suprathreshold color difference sensitivity.

### Suprathreshold color difference experiment

We measured sensitivities to suprathreshold color difference using MLDS. MLDS is a psychophysical method to estimate the psychological scale (perceptional magnitude) for a stimulus set whose properties differ along a single perceptual dimension based on the response probability of comparing perceptual differences between two paired stimuli.

### Stimulus

**Figure 12:**
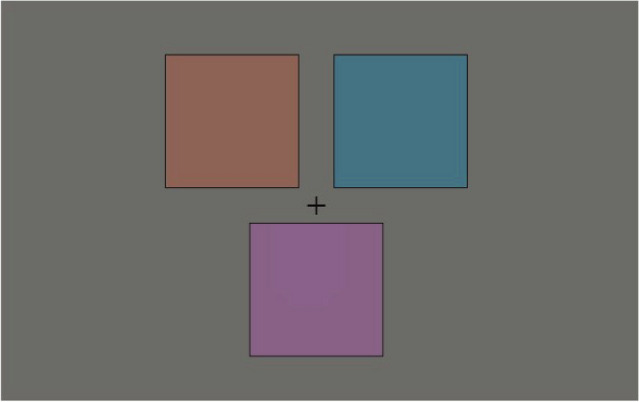
Stimulus in suprathreshold color difference experiment.

Figure 12 shows an example of the stimulus. The stimulus was a triplet of colored squares on a gray background at the screen center. Each square’s width and height were 2°, and the interval between the squares was 0.5°. All squares had 1-pixel black edges. The square at the bottom was the reference square, while the other two at the top were the test squares.

We used the same sample colors as the pedestal colors in the discrimination experiment for the square colors. In each trial, three square colors were randomly chosen from the 32 colors. Though the number of possible combinations of 3 out of 32 colors is 4960 = $$\left( {\begin{array}{*{20}c} {32} \\ 3 \\ \end{array} } \right)$$, we used only those that met the following criteria. We refer to the hue angle of the reference square as *B*, and those of the test squares as *A* and *C*. Color combinations with $\left| {A - B} \right|$ or $\left| {B - C} \right|$ greater than 90° were excluded from the stimuli because they were too difficult for the observer to judge. In addition, color combinations with $\left| \right|A - B\left| { - } \right|B - C\left| \right|$ greater than 25° were also excluded from the stimuli because the judgments were too easy. As a result, we used 1088 combinations.

### Procedure

The stimulus was presented for 1000 ms. The sample colors and positions of the two test squares (left or right) were determined randomly per trial. The squares constituted two pairs—left test/reference pair and right test/reference pair. The observer indicated which test/reference pair had a larger color difference in a two-alternative forced-choice (2AFC) manner by pressing one of two keys corresponding to left and right. The subsequent trial started 1000 ms after the observer’s response.

Each color combination was used in four trials, resulting in 4352 trials (= 1088 combinations × 4 repeats) in total. These trials were divided into eight sessions, each with 544 trials and lasting approximately 20 min. The order of color combinations was determined randomly, but every color combination was used only once in two sessions. The observer took a short break between sessions. At the beginning of each session, 30 practice trials with randomly chosen color combinations were conducted.

### Analysis

MLDS assumes that color difference perception is governed by an underlying color representation. We analyzed our response data and found that the perceptions of different magnitudes of color difference exhibit distinct internal sensitivity profiles, as shown in Fig. 2. Therefore, we computed distinct perceptual scales for small and large magnitudes of color difference by dividing the experimental data into smaller and larger magnitudes of color differences.

**Figure 13:**
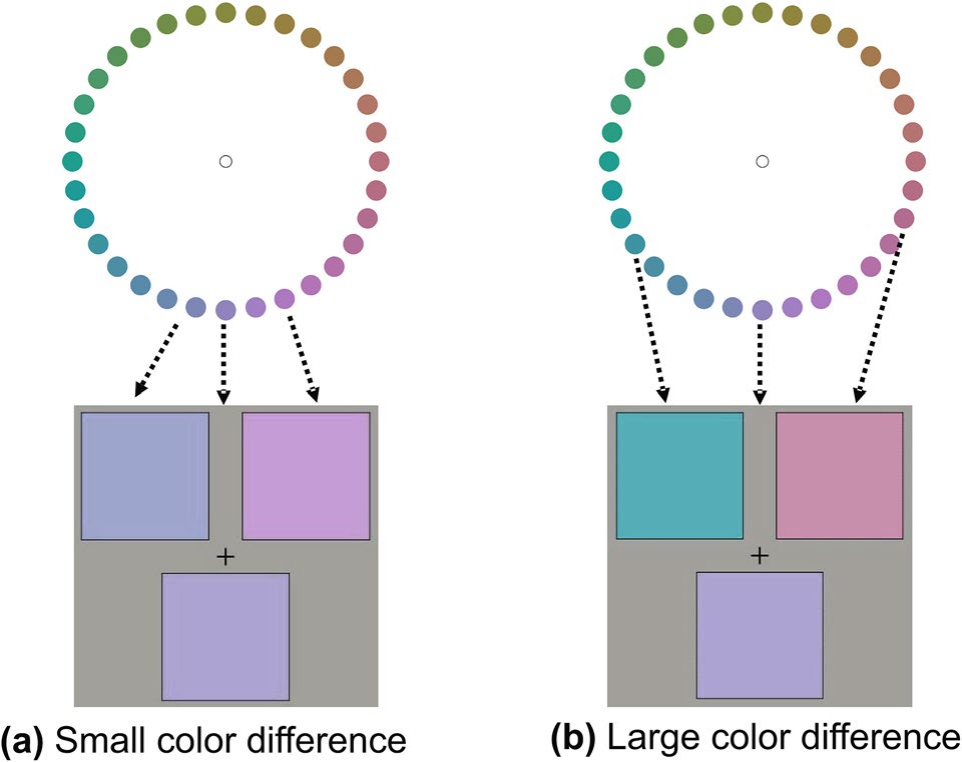
Example of color combinations with (**a**) small and (**b**) large color differences. The colors indicated by the arrows are used in the stimuli.

Color combinations with small color differences were defined as those with hue angle differences in the range of [11.25°, 45°], whereas color combinations with large color differences were those in [56.25°, 90°]. Illustrative examples of small and large color difference combinations are shown in Fig. 13.

The following steps were executed separately for small and large color differences.

1. Since the number of trials for each stimuli combination for one observer was only four, which was not enough to make an accurate calculation of perceptual scales, we integrated the responses from all seven observers (28 trials in total).
2. Based on the observers’ responses, we calculated the perceptual scales of all 32 sample colors using a customized function of *PAL_MLDS_Fit* and *PAL_MLDS_Bootstrap* in Palamedes Toolbox. In this model, the internal noise of the observers’ decisions was also estimated, as well as the perceptual scales.
3. The sensitivities were calculated by taking the differences in the perceptual scales between adjacent colors.
4. Finally, to compare sensitivity profiles among different sizes of color differences, the sensitivities were normalized by dividing them by mean sensitivity across the sample colors.

### Color category experiment

To investigate the relationship between the color difference sensitivities and color categories, we measured color category boundaries between the eight basic colors: red, yellow, green, blue, orange, purple, brown, and pink. Linguistically, basic color terms are defined as the elementary color terms that are universally used for communicating colors^23,37^. Witzel and Gegenfurtner^3^ similarly utilized these basic colors to examine the relationship between color discrimination and color categories.

### Stimulus

The stimulus layout was the same as that of suprathreshold color differences experiments shown in Fig. 12. All squares had an identical color chosen from 64 colors sampled at equal intervals on the hue circle (Fig. 9).

### Procedure

**Figure 14:**
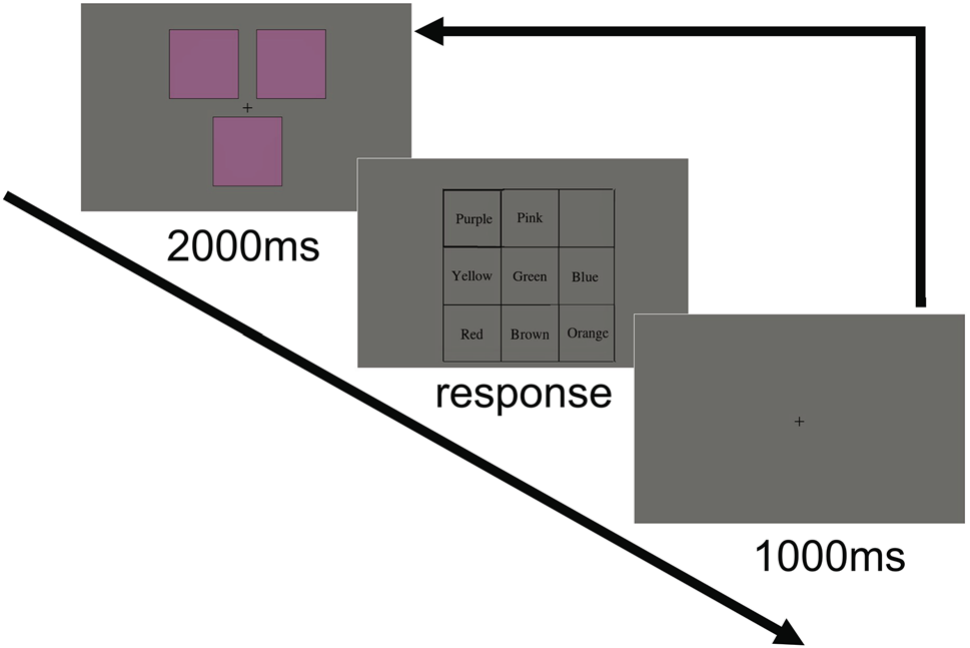
Stimulus presentation sequence in color category experiment.

Figure 14 illustrates the stimulus presentation sequence. In each trial, the three squares with the same color were presented to the observer for 2000 ms. The observer had to indicate the perceived basic color in an eight-alternative forced-choice (8AFC) manner by pressing one of eight keys corresponding to the eight basic colors. Five trials were conducted for each sample color, totaling 320 trials (= 64 colors × 5 trials). All trials were conducted within a single session lasting approximately 20 min. At the beginning of the session, the observer completed 20 practice trials with randomly selected sample colors.

It should be noted that only six of the eight basic color categories (pink, orange, yellow, green, blue, and purple, except for red and brown) were used by the observers. This could be attributed to the stimulus luminance (20 cd/m^2^), which was isoluminant with the background; under this luminance condition, red hues appear pink, and brown hues appear orange or yellow^38^.

### Analysis

We pooled the responses from all observers and estimated the hue angles of the category boundaries as follows. First, for each sample color, we calculated the response rate of each basic category. Then, we calculated category boundaries as follows. We defined $A_{down}$ as the hue angle of a sample color where the response rate for category A decreased from > 0.5 to < 0.5 and $B_{up}$ as the hue angle where the response rate for category B increased from < 0.5 to > 0.5. In most cases, $A_{down}$ and $B_{up}$ were equal, and we defined the category boundary as $A_{down} \left( { = B_{up} } \right)$. However, sometimes $A_{down}$ and $B_{up}$ were close but not equal, such as when three categories were used for a single sample color. In these cases, we assumed that $A_{up}$ and $B_{down}$, whose difference was less than 16.875° (= 360° × 3/64), formed a category boundary since this criterion seemed to capture the category boundaries correctly in our results in a preliminary analysis. We then defined the category boundary as $(A_{down} + B_{up} )/2$ in these cases.

## Data Availability

The datasets generated for this study are available on request to the corresponding author.
